# Comparative Performance of Large Language Models on European Gastroenterology Board-Style Questions: Analysis of Reasoning Versus Non-Reasoning Architectures

**DOI:** 10.3390/jcm15072692

**Published:** 2026-04-02

**Authors:** Cem Simsek, Petr Vanek, Hakan Aydinli, Jan Krivinka, Manuel Lehner, Sara Schiavone, Cesare Hassan, Henriette H. Heinrich

**Affiliations:** 1 Division of Gastroenterology, Hacettepe University, 06230 Ankara, Turkey; 2 Faculty of Medicine and Dentistry, Palacký University Olomouc, 77515 Olomouc, Czech Republic; 3 Department of Gastroenterology and Digestive Endoscopy, Masaryk Memorial Cancer Institute, 65653 Brno, Czech Republic; 4 Hepatology and Nutrition, Division of Gastroenterology, University of Minnesota, Minneapolis, MN 55455, USA; 5 2nd Department of Internal Medicine—Gastroenterology and Geriatrics, University Hospital Olomouc, Faculty of Medicine and Dentistry, Palacký University Olomouc, 77900 Olomouc, Czech Republic; 6 Department of Surgery, University Hospital Brno, Faculty of Medicine, Masaryk University, 62500 Brno, Czech Republic; 7 Gastroenterology, Clarunis Universitäres Bauchzentrum, 4058 Basel, Switzerland; 8 IRCCS Humanitas Research Hospital, 20089 Milan, Italy; 9 Department of Biomedical Sciences, Humanitas University, 20072 Milan, Italy

**Keywords:** artificial intelligence, ChatGPT, large language models, medical examination, ESEGH, EBGH, gastroenterology

## Abstract

**Background**: While large language models (LLMs) have demonstrated proficiency in medical examinations, their comparative performance on European gastroenterology assessments remains underexplored, particularly regarding architectural differences between reasoning and non-reasoning models. This study benchmarks five state-of-the-art LLMs—DeepSeek-R1, ChatGPT-o1, ChatGPT-4o, Gemini-1.5-Pro, and Llama-3.1-405B (All versions January 2025)—using 203 board-style questions from validated ESEGH preparation materials. **Methods**: Questions from two commercial ESEGH preparation banks were administered five times per model using standardized prompts. Accuracy, consistency, and domain-specific performance across clinical, diagnostic, and therapeutic questions were analyzed. Four practicing gastroenterologists validated human performance under uniform conditions. **Results**: ChatGPT-o1 achieved the highest overall accuracy at 84.0% (95% CI: 81.8–86.3), followed closely by ChatGPT-4o (81.7%), DeepSeek-R1 (79.0%), and Llama-3.1-405B (77.2%), while Gemini-1.5-Pro significantly underperformed with 68.5% accuracy (difference vs. ChatGPT-o1: 15.5 percentage points, 95% CI: 11.9 to 19.1, *p* < 0.01). Although all models exhibited high internal consistency ≥98.4% average agreement across repeated attempts, with 94.6–98.0% of questions answered identically in all five attempts), greater consistency did not necessarily correspond to higher accuracy. Domain-specific analysis revealed that diagnostic questions were answered most accurately, whereas clinical examination questions posed considerable challenges. Topic analysis demonstrated that questions on small intestine disorders were answered with the highest accuracy, in contrast to the lower performance observed in bariatric and pancreatic disorders. Notably, reasoning models, which employed explicit chain-of-thought strategies, outperformed non-reasoning counterparts (81.5% vs. 75.8%, difference: 5.7 percentage points, 95% CI: 3.4 to 8.0, *p* < 0.001), particularly on therapy questions and complex bait-and-switch formats. Practicing gastroenterologists achieved substantially lower accuracy (mean: 50.9%, range: 37.9–69.0%) compared to all LLMs. All models exceeded the current ESEGH passing threshold of 61.5%, with the top four models surpassing this benchmark by 15.7–22.5 percentage points. **Conclusions**: This benchmarking study demonstrates that current LLMs, particularly those with reasoning architectures, achieve high accuracy on European gastroenterology board-style questions. However, significant performance gaps in specific domains highlight limitations that must be addressed before clinical application. These findings provide a baseline for evaluating LLM capabilities in European medical contexts.

## 1. Introduction

Large language models (LLMs) are artificial intelligence systems trained on vast text corpora to understand and generate human language through pattern recognition and statistical inference. These models, including OpenAI’s ChatGPT series, Google’s Gemini, and Meta’s Llama, have demonstrated increasing proficiency in medical knowledge tasks. Early studies demonstrated ChatGPT-4’s strong performance on general medical licensing exams such as the United States Medical Licensing Examination (USMLE) as well as the Japanese licensing examination, where it achieved a passing threshold across multiple steps [1,2,3]. However, the performance of LLMs in specialist medical examinations, particularly in gastroenterology, has been mixed [4].

Recent studies have examined LLM performance on gastroenterology-specific examinations with variable results. ChatGPT-4 failed to pass the American College of Gastroenterology (ACG) self-assessment test, scoring below the 70% threshold required for competency [5]. However, Chat GPT-3.5 and AI Perplexity scored >80% in answering questions based on the Italian residents’ gastroenterology exam [6]. These mixed results suggest that LLM performance may depend on examination format, regional guideline differences, and model architecture.

Recent innovations have produced two distinct categories of LLMs: reasoning models that employ explicit chain-of-thought processes to decompose complex problems into intermediate steps (e.g., DeepSeek-R1, ChatGPT-o1), and non-reasoning models that generate responses through direct inference without visible intermediate reasoning (e.g., ChatGPT-4o, Gemini-1.5-Pro, Llama-3.1-405B). While systematic reviews demonstrate progressive improvement in LLM performance on medical examinations [2], comparative analyses of these architectural approaches remain limited, particularly in European medical contexts.

The European Specialty Examination in Gastroenterology and Hepatology (ESEGH) is a mandatory, high-quality, knowledge-based exam for board certification in the UK and Switzerland [7]. The examination comprises 200 multiple-choice questions aligned with the European Blue Book Curriculum. This standardized format and broad content coverage make ESEGH-style questions an appropriate benchmark for evaluating LLM performance in European gastroenterology contexts.

This study addresses the gap in comparative architectural analysis by systematically benchmarking five current LLMs on questions designed to simulate ESEGH content and format. Our objectives are to: (1) compare performance between reasoning and non-reasoning model architectures, (2) identify domain-specific performance patterns across clinical areas, and (3) establish baseline performance metrics for future studies.

## 2. Methods

### 2.1. Study Design

**Figure 1:**
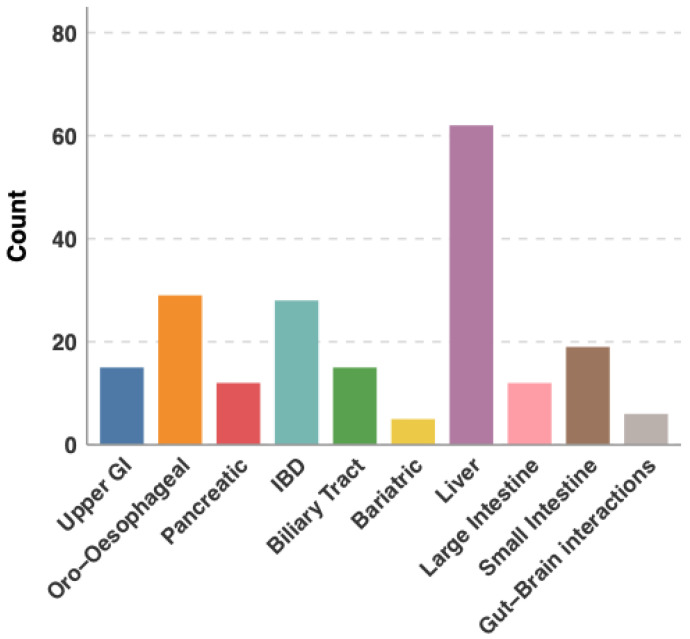
Question topics distribution.

**Figure 2:**
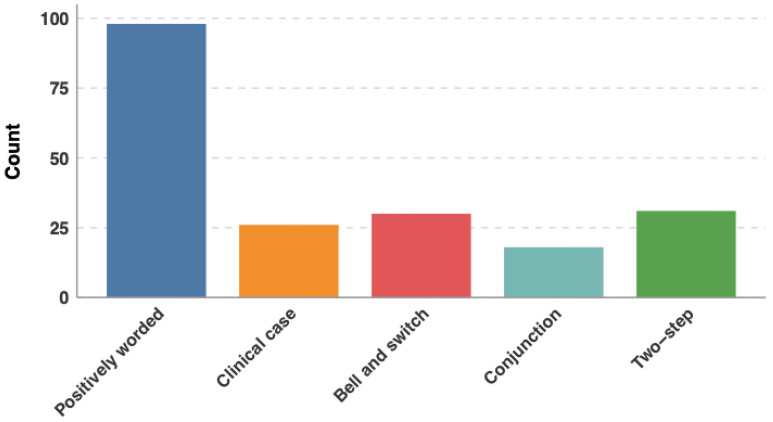
Question-type distribution.

This study evaluated the performance of five state-of-the-art LLMs on board-style questions designed to simulate ESEGH content and format. We employed a prospective, comparative design throughout the study period to assess LLM accuracy, consistency, and performance across different question types (Figure 1 and Figure 2) and domains (Table 1). As official ESEGH questions are not publicly available, the final dataset comprised 203 text-based multiple-choice questions selected to match the domain distribution of the official ESEGH blueprint and based on official question banks. A total of 203 questions were selected to simulate the 200-question ESEGH exam. Image-based questions were excluded due to variable image-processing capabilities across models (Clinical trial number: not applicable).

### 2.2. Question Selection and Classification

Questions were sourced from two commercial ESEGH preparation banks. To ensure content validity, two European Board of Gastroenterology and Hepatology (EBGH)-certified gastroenterologists independently evaluated questions for clinical accuracy, currency, and alignment with the ESEGH blueprint. From the pool, a board-certified gastroenterologist (C.S.) selected 203 questions to match the proportional distribution of the official ESEGH examination across European Blue Book Curriculum domains. Questions were classified by clinical domain (therapy, diagnosis, prognosis, etiology/harm, prevention, clinical examination), structural type (single-best answer, clinical vignette, two-step reasoning, bait-and-switch, conjunction), and curriculum topic according to the European Blue Book categories (Figure 1 and Table 1).

### 2.3. Large Language Models and Technical Specifications

We evaluated five state-of-the-art models, categorized into two groups based on their observable output behavior under standardized prompting. This classification represents an operational distinction rather than a strict architectural boundary, as all large language models perform some degree of implicit reasoning during inference. Models classified as ‘reasoning models’ are those that generate explicit, visible chain-of-thought processes to decompose problems into intermediate steps before arriving at a final answer. These included DeepSeek-R1 (DeepSeek AI) was released in December 2023, with 67B parameters (version 2.1.0). ChatGPT-o1 (OpenAI) was released in September 2023, with parameters not publicly disclosed (model version 2023-09-05-preview). Non-reasoning models, which generate responses through direct inference without visible intermediate reasoning, ChatGPT-4o (OpenAI) was released in May 2023 (version 0504), Gemini-1.5-Pro (Google) was released in January 2024 (version stable-1.5-pro-001), and Llama-3.1-405B (Meta) was released in April 2023, with 70B parameters (version 2.0). All models were accessed through their respective official APIs between January 2024 and 15 February 2025, using the most stable versions available during this period.

### 2.4. Question Administration Protocol

Following a pilot test with 20 questions (not included in the final analysis), a standardized protocol was developed for question administration. The pilot phase established that five repetitions per question optimally balanced consistency assessment with practical feasibility, as response patterns stabilized after 3–4 attempts. Each question was presented to each LLM five times to assess consistency and reliability. Response collection was manually performed by a researcher (H.A.). Questions were delivered using a consistent prompt template that instructed the model to:

*“You are taking a medical board examination in gastroenterology and hepatology. Please answer the following multiple-choice question by selecting the single best answer from options A through E. Provide the letter corresponding to your answer.”*

Questions were presented with minimum 1-min intervals after refreshing the model’s memory between attempts to ensure independence of responses and mitigate potential model state retention. This study was exempt from ethical review as it did not involve patient data or human subjects.

### 2.5. Human Expert Validation

To provide a preliminary clinical reference for contextualizing LLM performance, we recruited a convenience sample of four practicing gastroenterologists: two board-certified experts who had previously passed the ESEGH examination and two gastroenterology fellows (novices) who had not yet taken or studied for the examination. This exploratory comparison was designed to offer initial context rather than serve as a definitive human benchmark. All participants answered the same 203-question set under standardized conditions without time constraints or external resources.

### 2.6. Statistical Analysis and Performance Metrics

Statistical analyses were performed using R version 4.2.1 (R Foundation for Statistical Computing, Vienna, Austria) and Python 3.8 with specialized libraries (NumPy, Pandas, SciPy, versions Janury 2025). Consistency analysis employed Fleiss’ Kappa for inter-rater reliability among LLMs and pairwise agreement rates. Comparative analyses utilized chi-square tests for overall differences between categories, with Fisher’s exact tests for pairwise comparisons when expected cell counts were less than 5. To control for multiple comparisons, we applied the Bonferroni correction with an adjusted significance threshold of *p* < 0.01 for post-hoc tests. Topic-specific and domain-specific analyses were conducted to evaluate performance patterns across different question categories.

## 3. Results

### 3.1. Overall LLM Performance

**Figure 3:**
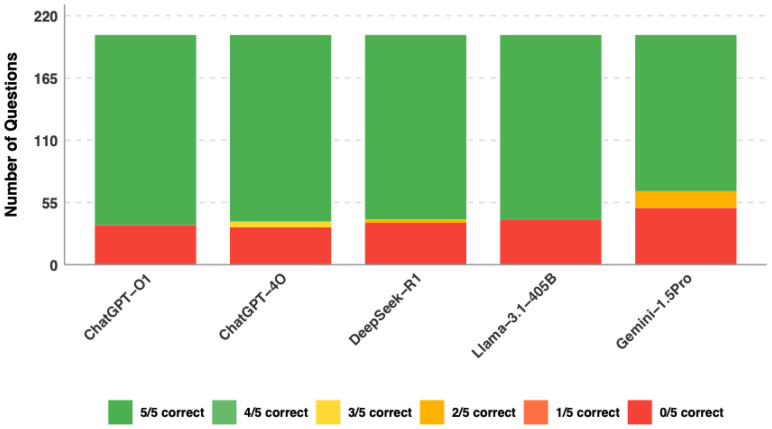
Distribution of correct answers by LLM (n = 203 questions).

**Figure 4:**
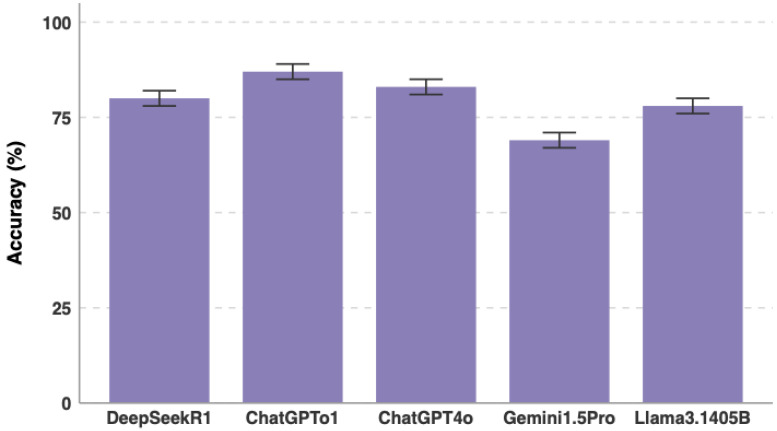
LLM Performance analysis.

A total of 203 single-best-answer questions reflecting the EBGH curriculum were included (Table 1). All five models were evaluated on each question five times, resulting in 5075 total responses (203 questions × 5 attempts × 5 models). All models exceeded the current ESEGH passing threshold of 61.5%, with accuracy ranging from 68.5% to 84.0%. ChatGPT-o1 achieved the highest overall accuracy at 84.0% (95% CI: 81.8–86.3), followed by ChatGPT-4o (81.7%, 95% CI: 79.3–84.1), DeepSeek-R1 (79.0%, 95% CI: 76.5–81.5), Llama-3.1-405B (77.2%, 95% CI: 74.7–79.8), and Gemini-1.5-Pro (68.5%, 95% CI: 65.6–71.3) (Table 2, Figure 3 and Figure 4). Gemini-1.5-Pro significantly underperformed relative to all other models (difference vs. ChatGPT-o1: 15.5 percentage points, 95% CI: 11.9 to 19.1; difference vs. ChatGPT-4o: 13.2 percentage points, 95% CI: 9.5 to 16.9; difference vs. DeepSeek-R1: 10.5 percentage points, 95% CI: 6.7 to 14.3; difference vs. Llama-3.1-405B: 8.7 percentage points, 95% CI: 4.9 to 12.5; *p* < 0.01 for each comparison). Among the top four models, differences in overall accuracy were not statistically significant when analyzed on a per-question basis (*p* > 0.05).

Using the most frequent answer across all 25 responses (5 attempts per model × 5 models) yielded an overall accuracy of 84.2%—equivalent to ChatGPT-o1’s individual best performance (Table 3). The consensus accuracy within each model’s 5 attempts did not exceed that model’s individual best result, suggesting minimal complementary knowledge across models. Consensus agreement was strongly associated with accuracy (Table 4); questions with >90% agreement across all responses had a 92.4% accuracy, whereas those with 50–60% agreement had only 54.5% accuracy.

### 3.2. Consistency Analysis

**Figure 5:**
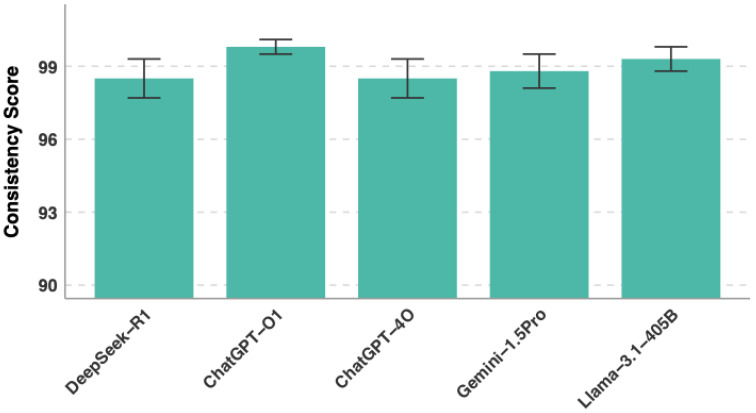
LLM Response consistency analysis.

All models displayed high internal consistency, measured by two complementary metrics (Table 5 and Table 6 and Figure 5). Average consistency (the mean agreement rate across all questions) ranged from 98.4% (ChatGPT-4o) to 99.6% (ChatGPT-o1). Full consistency (the percentage of questions with identical answers across all five attempts) ranged from 94.6% (DeepSeek-R1 and ChatGPT-4o) to 98.0% (ChatGPT-o1). ChatGPT-o1 led on both metrics, followed by Llama-3.1-405B (99.0% average, 96.6% full), Gemini-1.5-Pro (98.7% average, 95.6% full), DeepSeek-R1 (98.5% average, 94.6% full), and ChatGPT-4o (98.4% average, 94.6% full) (Table 4).

### 3.3. Performance by Clinical Domain

**Figure 6:**
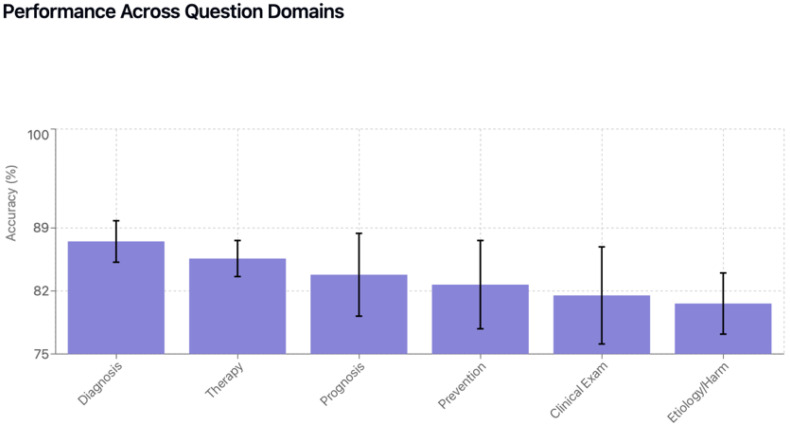
Performance across domains.

**Figure 7:**
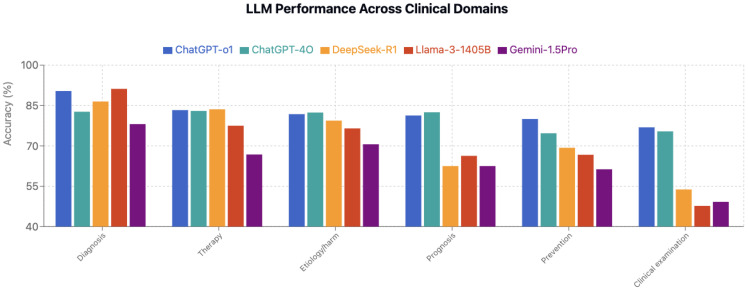
LLM Performance across clinical domains.

**Figure 8:**
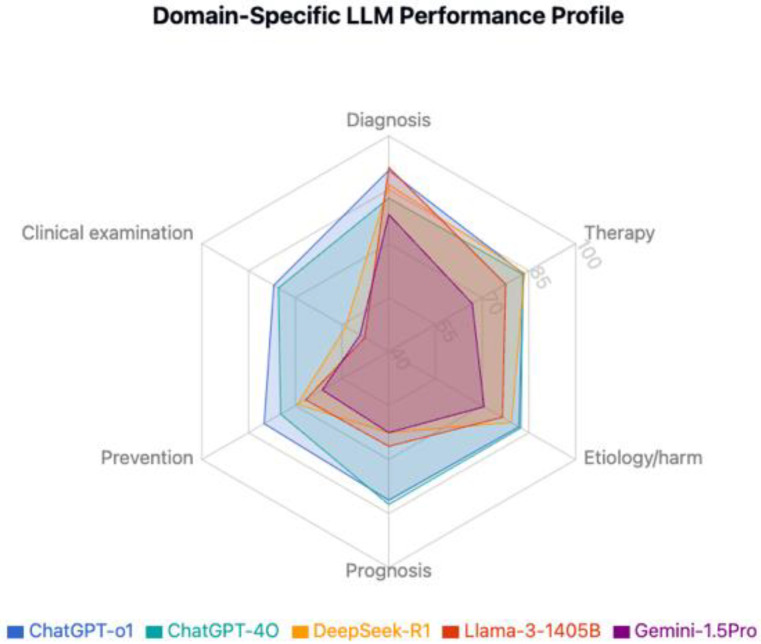
Domain-Specific LLM performance profile.

**Figure 9:**
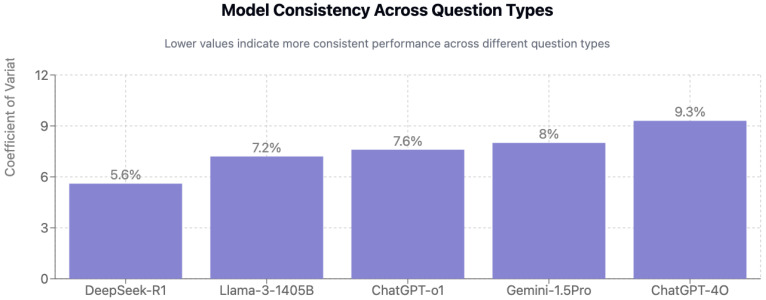
Model consistency across question types.

**Figure 10:**
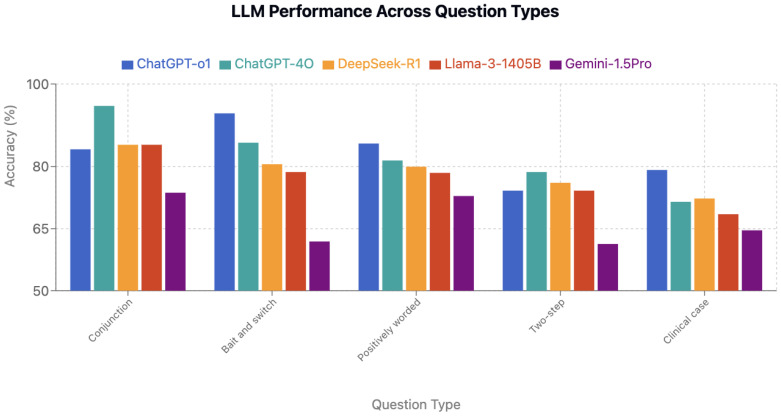
LLM performance across question types.

Paired model comparisons are presented in Table 7. Performance varied significantly across clinical domains. Clinical examination emerged as the most challenging domain (Table 8, Table 9, Table 10, Table 11 and Table 12, Figure 6, Figure 7 and Figure 8). ChatGPT-o1 (76.9%) and ChatGPT-4o (75.4%) performed significantly better than DeepSeek-R1 (53.8%), Llama-3.1-405B (47.7%), and Gemini-1.5-Pro (49.2%) in clinical examination questions (*p* < 0.01) (Table 9). Diagnosis was the highest-performing domain overall (85.8% average accuracy), with Llama-3.1-405B (91.2%) and ChatGPT-o1 (90.4%) notably scoring better. Question-type performance patterns are illustrated in Figure 9 and Figure 10**.**

Among the top four models, ChatGPT-4o demonstrated the most balanced performance (coefficient of variation [CV] = 4.3%), while Llama-3.1-405B showed the largest variability (CV = 21.3%) (Table 10), particularly strong in diagnosis (91.2%) but weak in clinical examination (47.7%) (Table 8). Within-model comparisons highlighted four of five models performed significantly worse on clinical examination than on their strongest domain (*p* < 0.05 for each); ChatGPT-4o was the only model without significant domain-based performance gaps (*p* = 0.236) (Table 11).

### 3.4. Performance by Question Topic

**Figure 11:**
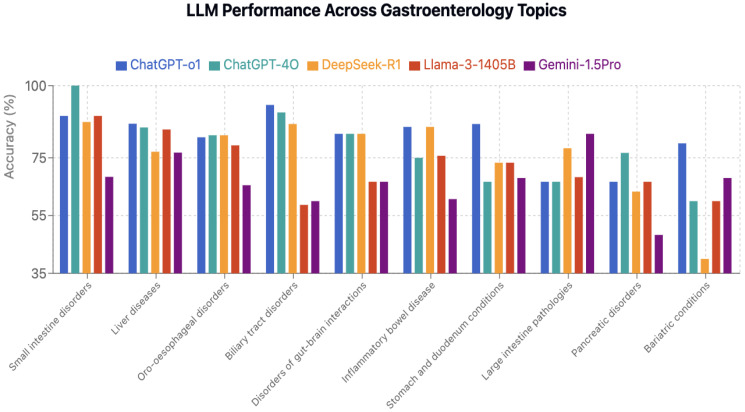

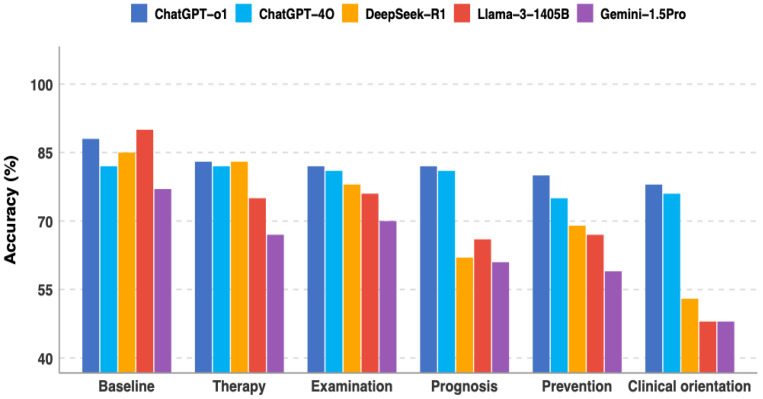
LLM performance across gastroenterology topics.

**Figure 12:**
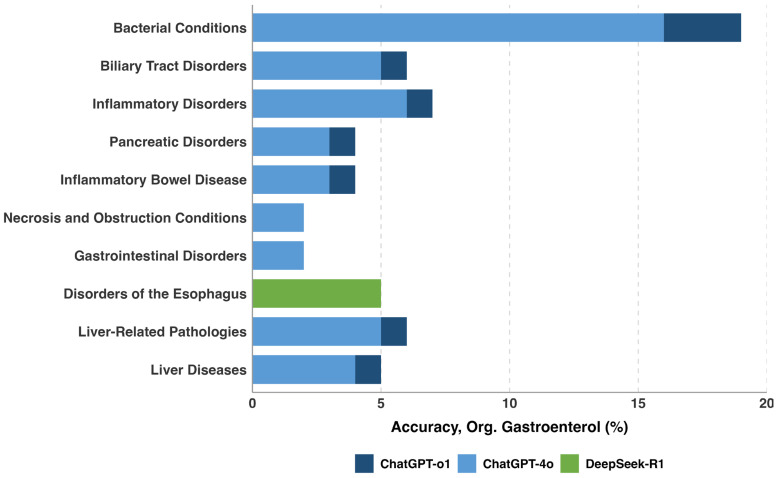
Performance gap between best and worst models by topic.

**Figure 13:**
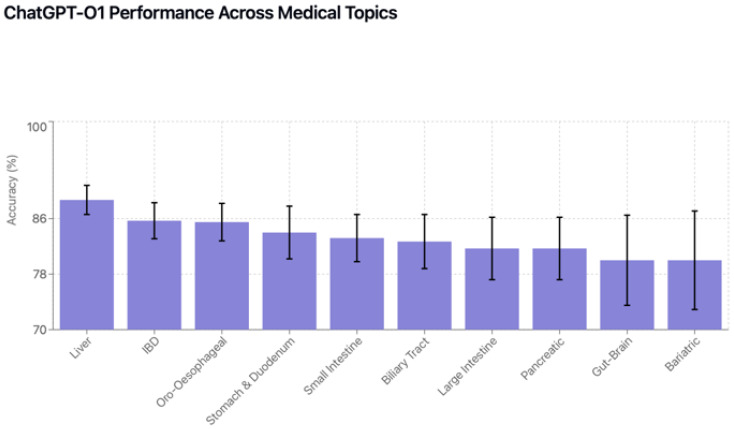
ChatGPT-o1 performance across medical topics.

Topic-specific analysis revealed substantial performance variation across gastroenterology subspecialties (Table 13 and Table 14). Small intestine disorders had the highest average accuracy (86.9%), with ChatGPT-4o achieving perfect (100%) accuracy. Bariatric conditions and pancreatic disorders were most challenging overall (61.6% and 64.3% average accuracy, respectively) (Figure 11). All models had statistically significant best–worst topic gaps (*p* < 0.001 in each model), ranging from 26.7 percentage points (ChatGPT-o1) to 47.4 points (DeepSeek-R1) (Figure 12).ChatGPT-o1 demonstrated consistent performance across most gastroenterology topics, as illustrated in Figure 13. Each model exhibited unique topic-specific strengths (Table 15). ChatGPT-o1 performed best in biliary tract disorders (93.3%). Llama-3.1-405B excelled at small intestine (%89,5) but struggled with biliary tract questions (58.7%). Gemini-1.5-Pro was notably better on large intestine pathologies (83.3%) than most other topics. 

### 3.5. Performance by Question Type

Conjunction questions (n = 19) were easiest overall (84.6% average), whereas clinical case questions (n = 26) showed the lowest accuracy (71.2%) (Table 1 and Figure 9). ChatGPT-o1 excelled at bait-and-switch questions (92.9%), while ChatGPT-4o led on conjunction questions (94.7%) (Figure 10). DeepSeek-R1 displayed the most consistent performance across types (CV = 5.6%). In contrast, ChatGPT-4o showed the widest question-type variation (23.2 percentage-point range, *p* < 0.05 for multiple within-model comparisons).

### 3.6. Reasoning Versus Non-Reasoning Models

**Figure 14:**
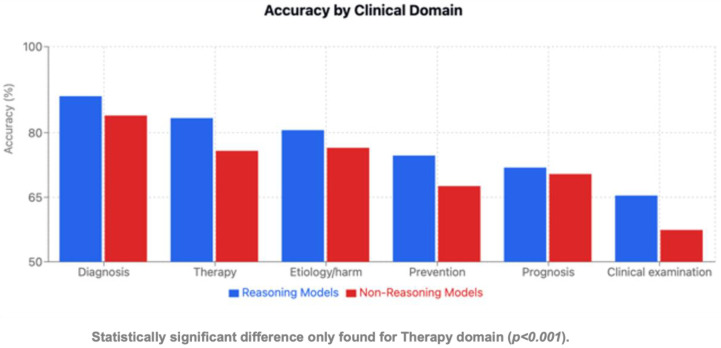
Accuracy by clinical domain.

**Figure 15:**
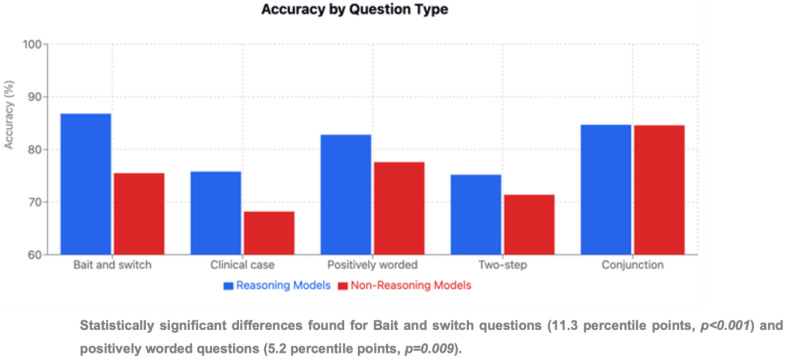
Accuracy by question type.

**Figure 16:**
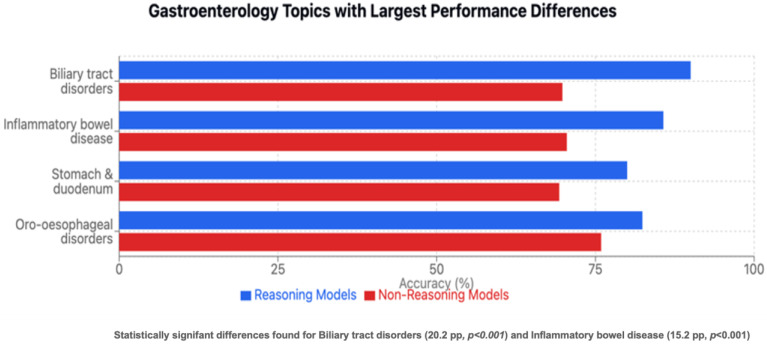
Gastroenterology topic with the largest performance differences.

**Figure 17:**
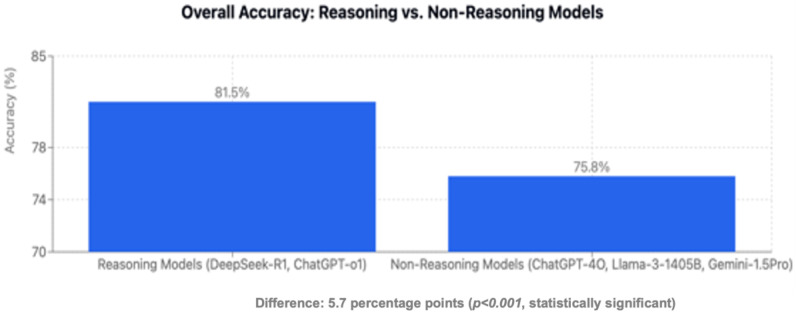
Overall accuracy reasoning vs. non-reasoning models.

Domain-specific analyses revealed significant advantages for reasoning models in therapy questions (83.4% vs. 75.8%, *p* < 0.001), while differences in other types did not reach statistical significance (Figure 14). When examining question types, reasoning models demonstrated significant advantages in bait-and-switch questions (86.8% vs. 75.5%, 95% CI: 3.9 to 11.3, *p* < 0.001) and positively worded questions (82.8% vs. 77.6%, 95% CI: 2.0 to 8.4, *p* = 0.009) (Figure 15). The most pronounced differences appeared in specific gastroenterology topics: biliary tract disorders (90.0% vs. 69.8%, 20.2 percentage points, 95% CI: 12.5 to 27.9, *p* < 0.001) and inflammatory bowel disease (85.7% vs. 70.5%, difference: 15.2 percentage points, 95% CI: 9.2 to 21.2, *p* < 0.001) (Figure 16). Reasoning LLMs (DeepSeek-R1, ChatGPT-o1) significantly outperformed non-reasoning models (ChatGPT-4o, Llama-3.1-405B, Gemini-1.5-Pro) with an overall accuracy of 81.5% vs. 75.8% (difference: 5.7 percentage points; 95% CI: 3.4 to 8.0, *p* < 0.001) (Figure 17). No domains or question types showed significant advantages for non-reasoning models.

### 3.7. Human Performance Reference

**Figure 18:**
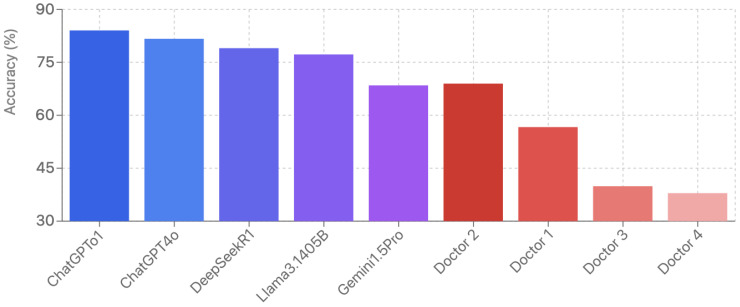
Performance comparison of medical doctors versus language models.

In our exploratory human reference sample, the four gastroenterologists demonstrated substantial performance variability, with overall accuracy ranging from 37.93% to 68.97% (mean: 50.86%, 95% CI: 27.47–74.26%). Expert physicians (mean: 62.81%) outperformed novice fellows (mean: 38.92%) by 23.89 percentage points (*p* < 0.05). Notably, all five LLMs significantly outperformed both expert and novice physicians, with the best-performing model (ChatGPT-o1: 84.04%) exceeding even the highest-scoring expert physicians by 15.07 percentage points. Inter-physician agreement was modest at 43.3%, substantially lower than the high consistency observed across LLM responses (>98.4%) (Figure 18). However, given the small and heterogeneous sample, these human results should be interpreted as preliminary contextual data rather than representative benchmarks of gastroenterologist performance.

## 4. Discussion

This benchmarking study provides the first systematic comparison of reasoning versus non-reasoning LLM architectures on European gastroenterology board-style questions. The key finding—that reasoning models outperformed non-reasoning models by 5.7 percentage points overall, with advantages exceeding 20 percentage points in specific domains—suggests that explicit chain-of-thought processing enhances performance on complex medical questions. All evaluated models exceeded the ESEGH passing threshold, with four of five achieving accuracy levels that would place them in the top performance tier of human test-takers. However, substantial variation across clinical domains and question types reveals important limitations that must be understood before considering any clinical applications.

Our findings align with a rapidly growing body of research on the use of LLMs in medical education and specialty board exams. Many earlier investigations focused on the ability of models on diverse medical licensing examinations. Ali et al. [8] reported that GPT-4 achieved scores comparable to human test-takers on the ACG self-assessment exams, scoring 76.3% on a text-based question set. Interestingly, the average human examinee scored 75.7%, suggesting near-equal performance between GPT-4 and board-eligible gastroenterologists. Safavi-Naini et al. (2024) compared GPT-4o and Claude-3.5-Sonnet with Llama and Mistral on gastroenterology exams [9]. They found that GPT-4o and Claude-3.5-Sonnet achieved the highest accuracy (73.7–74.0%). Samaan et al. [10] demonstrated that advanced prompt engineering strategies such as Retrieval-Augmented Generation (RAG) substantially improved GPT-4’s performance on specialty gastroenterology exams—from 60.3% to 80.7%.

The performance range observed in our study (68.5–84.0%) aligns with LLM evaluations across other medical specialties, where accuracies typically range from 60% to 89%. Gilson et al. [11] evaluated ChatGPT on USMLE questions, revealing passing or near-passing performance (over 60%). Ali et al. [12] and Chan et al. [13] showed that GPT-4 surpassed human pass marks in neurosurgery and MRCS Part A exams, respectively. Angel et al. [14] found that GPT-4 performed at 89% accuracy on the North American Veterinary Licensing Examination, exceeding GPT-3 and Bard. Other subfields beyond gastroenterology showed similar findings. For instance, Longwell et al. [15] demonstrated 84.4–86.7% correctness on ASCO and ESMO oncology questions. Schubert et al. [16] reported GPT-4 scoring 85.0% on neurology board-style examinations. Tarabanis et al. [17] noted GPT-4’s 77.5–80.7% performance on internal medicine board-style questions, occasionally surpassing human respondents. Our results fit within this performance range. While vision–language models have been proposed to address image-based questions, Safavi-Naini et al. [9] noted that LLMs often struggle with images unless a thorough human-crafted description is provided. We did not explore the image-interpretation, but existing evidence suggests that current multimodal approaches still lag behind text-based results.

An important consideration is the potential difference between the commercial preparation materials used in this study and the actual ESEGH examination questions. While the preparation banks we employed are designed to simulate the content, format, and difficulty of the official examination, several factors may limit direct generalizability. First, official ESEGH questions undergo rigorous psychometric validation, including item analysis and calibration against candidate performance data, which may result in more precisely calibrated difficulty levels and distractor effectiveness. Second, the official examination committee may employ specific question-writing conventions, clinical scenarios, or emphasis on emerging topics that are not fully captured in third-party materials. Third, the security of actual examination content means that preparation materials are necessarily approximations based on published curricula and candidate recall rather than direct replications. To establish definitive performance benchmarks, future research should pursue collaboration with the European Board of Gastroenterology and Hepatology to obtain access to validated examination items under appropriate confidentiality agreements.

A few studies highlight potential pitfalls. Koga et al. [18] found inconsistency and inaccuracies in LLM answers to pathology questions. Kaiser et al. [19] similarly reported incomplete or vague responses about colon cancer management in publicly available LLMs. Finally, Igarashi et al. [20] evaluated ChatGPT on Japanese emergency medicine board certification exams, finding 62.3% accuracy. This discrepancy may be due to differences in exam style, language, or localized guidelines.

The superior performance of reasoning models likely stems from their ability to decompose complex questions into intermediate steps, particularly evident in questions requiring the filtering of irrelevant information. It is important to note that our reasoning versus non-reasoning classification reflects observable output behavior under standardized prompting rather than fundamental architectural differences—all LLMs engage in some form of internal computation that could be considered reasoning. The 11.3 percentage-point advantage on bait-and-switch questions demonstrates this capability. However, all models showed weaknesses in bariatric (61.6%) and pancreatic disorders (64.3%), suggesting training data limitations rather than architectural constraints. These subspecialty gaps highlight that even advanced architectures cannot compensate for insufficient domain representation in training corpora.

A limitation of our analysis is that we did not systematically categorize the types of errors made by each model. Incorrect answers may stem from distinct failure modes, including outdated or missing medical knowledge, guideline mismatches between European and North American recommendations, flawed reasoning chains, or misinterpretation of question distractors. Understanding these failure modes is essential for targeted model improvement. Future studies should incorporate structured error taxonomies to distinguish knowledge deficits from reasoning failures, which would provide actionable insights for both model developers and clinical end-users.

Importantly, this study was not designed as a human-versus-machine competition. The primary contribution lies in the systematic comparison of reasoning versus non-reasoning LLM architectures on European gastroenterology content. The human reference data contextualizes these findings but does not constitute a powered comparison. Our exploratory human comparison provides preliminary context suggesting that LLMs may match physician performance on standardized board-style questions. However, several important caveats warrant emphasis. First, our convenience sample of four physicians—two board-certified experts and two fellows—was designed to provide initial reference points rather than establish definitive human benchmarks. The observed low inter-physician agreement (43.3%) likely reflects both the inherent complexity of specialty-level medical knowledge and the heterogeneity of our small sample, which intentionally spanned different expertise levels. A larger, more homogeneous cohort of ESEGH-certified gastroenterologists would be needed to establish robust human performance baselines. Second, the expert-novice performance gap (62.8% vs. 38.9%) aligns with expected expertise gradients, suggesting our sample captured meaningful variation despite its size. Third, standardized examinations assess only a subset of clinical competence; they do not capture diagnostic reasoning at the bedside, procedural skills, patient communication, or the integration of contextual factors that define expert clinical practice.

The finding that all LLMs exceeded individual physician scores should therefore not be interpreted as evidence that these models can replace clinical judgment. Future studies should incorporate larger physician cohorts, ideally stratified by years of experience and recent ESEGH examination performance, to establish more reliable human benchmarks against which LLM capabilities can be meaningfully assessed.

Data contamination is another consideration. The commercial preparation materials used in this study may exist in some models’ training data, which could inflate performance through memorization rather than reasoning. Several observations partially address this concern. The substantial variation in performance across models suggests contamination did not affect all models equally. Additionally, the consistent difficulty of clinical examination questions and bariatric topics across all models suggests reasoning rather than pure recall—memorized content would likely show more uniform performance. However, we cannot definitively exclude contamination without access to training data documentation, which model developers do not publicly disclose. Future studies should consider using newly developed or embargoed questions to minimize this risk.

Our study has several limitations. The board exam is not directly correlated with clinical skills, but instead reflects a selected knowledge base; thus, our findings should be interpreted primarily in an educational rather than clinical context. The questions used in this analysis were sourced from specialized ESEGH preparation materials rather than from actual past exams. While these high-quality mock questions approximate the real exam structure, the absence of original ESEGH items may affect generalizability—particularly if official questions differ in nuance, ambiguity, or distractor design. Additionally, image-based items were excluded due to variable multimodal capabilities across models. These questions, which represent a notable subset of ESEGH content, may pose distinct challenges for current LLMs. The lack of direct benchmarking against human test-takers also prevents practical conclusions about clinical utility or deployment readiness. While the ESEGH multiple-choice format provides a useful framework for comparison, it remains a simplified abstraction of complex medical reasoning that may artificially inflate model performance. Nonetheless, the top-performing models in our study achieved scores exceeding the published pass rates for recent ESEGH sittings, suggesting potential utility as board-preparation tools. The human reference sample was small (n = 4) and heterogeneous by design, limiting the generalizability of human-LLM comparisons. This exploratory comparison should be replicated with larger, more homogeneous physician cohorts before drawing conclusions about relative human-AI performance. We did not perform a detailed error analysis to distinguish between different failure modes, such as knowledge gaps, reasoning errors, or guideline mismatches. Such an analysis would provide valuable insights into the specific weaknesses of each model and inform targeted improvements.

Our findings demonstrate that the top-performing LLMs achieved accuracy levels exceeding current ESEGH examination standards. With ChatGPT-o1 reaching 84.0% accuracy and the top four models all scoring above 77%, these results surpass both the historical pass threshold of 59% (2019) and the current equated pass mark of 61.5% (2022 onwards). The 432-point equated score corresponds to 61.5% accuracy, meaning that all evaluated LLMs would achieve passing scores. However, these findings should be interpreted within the context that our study used preparation materials rather than actual ESEGH questions, and real examination conditions may present additional challenges not captured in our assessment. The substantial margin by which LLMs exceeded the passing threshold suggests potential utility as study aids for candidates preparing for the ESEGH, though the clinical relevance of this performance advantage requires further investigation in real-world educational settings. Our sample size of 203 questions may underrepresent certain subspecialties and rare conditions. Focusing exclusively on European guidelines also limits generalizability to other healthcare systems and regional standards. We evaluated only a narrow slice of model functionality without assessing their ability to provide reasoning rationales. Our standardized prompting approach likewise did not explore the full impact of prompt engineering, which may significantly influence model performance. Finally, we did not analyze the underlying causes of incorrect answers—such as outdated knowledge, guideline mismatch, or distractor misinterpretation—each of which could inform future model refinement. Given these limitations, our findings should be interpreted cautiously as an initial exploration rather than evidence of clinical capability.

This benchmarking study establishes baseline performance metrics for current LLM architectures on European gastroenterology board-style questions. The consistent advantage of reasoning models suggests that future development should prioritize architectures capable of explicit problem decomposition. However, persistent weaknesses in clinical examination questions and subspecialized topics indicate fundamental limitations that architectural improvements alone cannot address. Future research should: (1) validate these findings using official examination materials through collaboration with the EBGH, (2) investigate whether reasoning advantages translate to other medical specialties, (3) develop multimodal capabilities for image-based questions, (4) perform detailed error analyses to distinguish knowledge deficits from reasoning failures and guideline mismatches, and (5) most importantly, assess whether high test performance correlates with any clinically meaningful outcomes. Until such evidence exists, LLMs should be viewed as emerging technologies requiring rigorous evaluation rather than ready tools for medical education or practice.

## Figures and Tables

**Table 1:** Characteristics of ESEGH questions (N = 203).

Characteristic	n (%)
**Question Topic**	
Liver diseases	62 (30.5)
Oro-esophageal disorders	29 (14.3)
Inflammatory bowel disease	28 (13.8)
Small intestine disorders	19 (9.4)
Stomach and duodenum conditions	15 (7.4)
Biliary tract disorders	15 (7.4)
Pancreatic disorders	12 (5.9)
Large intestine pathologies	12 (5.9)
Disorders of gut-brain interactions	6 (3.0)
Bariatric conditions	5 (2.5)
Question Type	
Positively worded questions	96 (47.3)
Two-step questions	31 (15.3)
Bait-and-switch questions	31 (15.3)
Clinical case questions	26 (12.8)
Conjunction questions	19 (9.4)
Knowledge Evidence Base	
Expert opinion	79 (38.9)
Multiple guidelines	59 (29.1)
Clinical practice	47 (23.2)
Single guideline	18 (8.9)
Clinical Domain	
Therapy	73 (36.0)
Diagnosis	52 (25.6)
Etiology/harm	34 (16.7)
Prognosis	16 (7.9)
Prevention	15 (7.4)
Clinical examination	13 (6.4)

**Table 2:** Overall LLM accuracy on ESEGH questions.

LLM Model	Accuracy, % (95% CI)	Correct Answers/Total Attempts
ChatGPT-o1	84.0 (81.8–86.3)	853/1015
ChatGPT-4o	81.7 (79.3–84.1)	829/1015
DeepSeek-R1	79.0 (76.5–81.5)	802/1015
Llama-3.1-405B	77.2 (74.7–79.8)	784/1015
Gemini-1.5-Pro	68.5 (65.6–71.3)	695/1015

Note: Total attempts refer to 5 attempts for every 203 questions (5 × 203).

**Table 3:** LLM Accuracy using a consensus-based approach.

Model Approach	Accuracy, % (95% CI)	Correct Consensus Answers/Total Questions
Cross-LLM Consensus	84.2 (79.2–89.2)	171/203
ChatGPT-o1 consensus	84.2 (79.2–89.2)	171/203
ChatGPT-4o consensus	81.3 (75.9–86.6)	165/203
DeepSeek-R1 consensus	79.3 (73.7–84.9)	161/203
Llama-3.1-405B consensus	77.8 (72.1–83.5)	158/203
Gemini-1.5-Pro consensus	68.0 (61.6–74.4)	138/203

Note: “Consensus” approaches use the most frequent answer across all 5 attempts for each question. “Cross-LLM Consensus” uses the most frequent answer across all 25 responses (5 attempts × 5 models) for each question.

**Table 4:** Relationship between consensus agreement level and accuracy.

Consensus Agreement	Accuracy, %	Correct Consensus/Total Questions	Percentage of Questions
90–100%	92.4	109/118	58.1%
80–90%	82.9	34/41	20.2%
70–80%	83.3	5/6	3.0%
60–70%	63.0	17/27	13.3%
50–60%	54.5	6/11	5.4%
**Overall**	84.2	171/203	**100%**

Note: Consensus agreement refers to the percentage of all 25 responses (across all models and attempts) that agreed on the most common answer.

**Table 5:** LLM Consistency analysis overall consistency metrics.

Model	Average Consistency (%)	Fully Consistent Questions (%)	Fully Consistent and Correct Questions (%)
ChatGPT-o1	99.6	98.0 (199/203)	82.8 (168/203)
Llama-3.1-405B	99.0	96.6 (196/203)	75.4 (153/203)
Gemini-1.5-Pro	98.7	95.6 (194/203)	66.5 (135/203)
DeepSeek-R1	98.5	94.6 (192/203)	76.8 (156/203)
ChatGPT-4o	98.4	94.6 (192/203)	80.3 (163/203)

Note: Fully consistent questions refers to 5/5 agreement over 5 attempts for each question.

**Table 6:** Consistency distribution by level.

Model	100% Consistency	80–99% Consistency	60–79% Consistency	40–59% Consistency	20–39% Consistency
ChatGPT-o1	98.0% (199)	2.0% (4)	0.0% (0)	0.0% (0)	0.0% (0)
Llama-3.1-405B	96.6% (196)	2.0% (4)	1.5% (3)	0.0% (0)	0.0% (0)
Gemini-1.5-Pro	95.6% (194)	2.5% (5)	2.0% (4)	0.0% (0)	0.0% (0)
DeepSeek-R1	94.6% (192)	3.4% (7)	2.0% (4)	0.0% (0)	0.0% (0)
ChatGPT-4o	94.6% (192)	3.0% (6)	2.5% (5)	0.0% (0)	0.0% (0)

**Table 7:** Paired model comparisons.

Model 1	Model 2	Model 1 Only Correct	Model 2 Only Correct	Both Correct	Both Wrong	*p*-Value
ChatGPT-o1	ChatGPT-4o	15	9	156	23	0.532
ChatGPT-o1	DeepSeek-R1	22	12	149	20	0.265
ChatGPT-o1	Llama-3.1-405B	23	10	148	22	0.094
ChatGPT-o1	Gemini-1.5-Pro	39	6	132	26	<0.001
ChatGPT-4o	DeepSeek-R1	19	15	146	23	0.835
ChatGPT-4o	Llama-3.1-405B	21	14	144	24	0.547
ChatGPT-4o	Gemini-1.5-Pro	36	9	129	29	<0.001
DeepSeek-R1	Llama-3.1-405B	19	16	142	26	0.915
DeepSeek-R1	Gemini-1.5-Pro	38	15	123	27	0.008
Llama-3.1-405B	Gemini-1.5-Pro	29	9	129	36	0.007

Note: McNemar’s test compares model performance on a question-by-question basis using the consensus answer from each model’s 5 attempts. This paired analysis accounts for the fact that some questions may be inherently more difficult than others. “Model 1 Only Correct” means the number of questions where only Model 1 gave the correct consensus answer, while Model 2 was incorrect.

**Table 8:** LLM accuracy by clinical domain.

Clinical Domain (n, %)	ChatGPT-o1	ChatGPT-4o	DeepSeek-R1	Llama-3.1-405B	Gemini-1.5-Pro
Therapy (73, 36.0%)	83.3 (79.5–87.1)	83.0 (79.2–86.9)	83.6 (79.8–87.4)	77.5 (73.3–81.8)	66.8 (62.0–71.7)
Diagnosis (52, 25.6%)	90.4 (86.8–94.0)	82.7 (78.1–87.3)	86.5 (82.4–90.7)	91.2 (87.7–94.6)	78.1 (73.0–83.1)
Prognosis (16, 7.9%)	81.3 (72.7–89.8)	82.5 (74.2–90.8)	62.5 (51.9–73.1)	66.3 (55.9–76.6)	62.5 (51.9–73.1)
Etiology/harm (34, 16.7%)	81.8 (76.0–87.6)	82.4 (76.6–88.1)	79.4 (73.3–85.5)	76.5 (70.1–82.8)	70.6 (63.7–77.4)
Prevention (15, 7.4%)	80.0 (70.9–89.1)	74.7 (64.8–84.5)	69.3 (58.9–79.8)	66.7 (56.0–77.3)	61.3 (50.3–72.4)
Clinical examination (13, 6.4%)	76.9 (66.7–87.2)	75.4 (64.9–85.9)	53.8 (41.7–66.0)	47.7 (35.5–59.8)	49.2 (37.1–61.4)
Overall (203, 100%)	84.0 (81.8–86.3)	81.7 (79.3–84.1)	79.0 (76.5–81.5)	77.2 (74.7–79.8)	68.5 (65.6–71.3)

Note: Values represent accuracy percentages with 95% confidence intervals in parentheses. No questions were in the Cost considerations domain.

**Table 9:** Pairwise model comparison—significant performance differences by domain.

Domain	Significant Performance Differences (*p* < 0.05)
Therapy	DeepSeekR1, ChatGPT-o1, ChatGPT-4o, Llama-3.1-405B > Gemini-1.5-Pro
Diagnosis	Llama-3.1-405B, ChatGPT-o1 > ChatGPT-4o, Gemini-1.5-Pro, DeepSeekR1 > Gemini-1.5Pro
Prognosis	ChatGPT-4o, ChatGPT-o1 > DeepSeekR1, Gemini-1.5-Pro
Etiology/harm	ChatGPT-4o > Gemini-1.5-Pro
Prevention	ChatGPT-o1 > Gemini-1.5-Pro
Clinical examination	ChatGPT-o1, ChatGPT-4o > DeepSeekR1, Gemini-1.5-Pro, Llama-3.1-405B

Note: Models on the left side of “>“ significantly outperform models on the right side for the specified domain.

**Table 10:** Performance variation across domains.

Model	Performance Range	Variation (%)	Strongest Domain	Weakest Domain
ChatGPT-o1	76.9–90.4%	5.9	Diagnosis (90.4%)	Clinical examination (76.9%)
ChatGPT-4o	74.7–83.0%	4.3	Prognosis (82.5%)	Prevention (74.7%)
DeepSeek-R1	53.8–86.5%	16.9	Diagnosis (86.5%)	Clinical examination (53.8%)
Llama-3.1-405B	47.7–91.2%	21.3	Diagnosis (91.2%)	Clinical examination (47.7%)
Gemini-1.5-Pro	49.2–78.1%	13.9	Diagnosis (78.1%)	Clinical examination (49.2%)

**Table 11:** Domain performance range for each model.

Model	Best Domain (Accuracy %)	Worst Domain (Accuracy %)	Difference (*p*)	*p*-Value	Significant?
ChatGPT-4o	Therapy (83.0%)	Prevention (74.7%)	8.3	0.236	No
ChatGPT-o1	Diagnosis (90.4%)	Clinical examination (76.9%)	13.5	0.013	Yes
DeepSeek-R1	Diagnosis (86.5%)	Clinical examination (53.8%)	32.7	<0.001	Yes
Gemini-1.5-Pro	Diagnosis (78.1%)	Clinical examination (49.2%)	28.8	<0.001	Yes
Llama-3.1-405B	Diagnosis (91.2%)	Clinical examination (47.7%)	43.5	<0.001	Yes

**Table 12:** Domain performance ranking for each model.

Domain	ChatGPT-o1	ChatGPT-4o	DeepSeek-R1	Llama-3.1-405B	Gemini-1.5-Pro
Diagnosis	1 (90.4%)	2 (82.7%)	1 (86.5%)	1 (91.2%)	1 (78.1%)
Therapy	2 (83.3%)	1 (83.0%)	2 (83.6%)	2 (77.5%)	3 (66.8%)
Etiology/harm	3 (81.8%)	4 (82.4%)	3 (79.4%)	3 (76.5%)	2 (70.6%)
Prognosis	4 (81.3%)	3 (82.5%)	5 (62.5%)	5 (66.3%)	4 (62.5%)
Prevention	5 (80.0%)	6 (74.7%)	4 (69.3%)	4 (66.7%)	5 (61.3%)
Clinical examination	6 (76.9%)	5 (75.4%)	6 (53.8%)	6 (47.7%)	6 (49.2%)
Coefficient of Variation	5.0%	4.5%	16.2%	18.8%	13.7%

Note: Numbers (1–6) represent the ranking of each domain within each model’s performance, with 1 being the highest. Percentages in parentheses show the accuracy for that domain. Coefficient of Variation (CV) measures the consistency of performance across domains, with lower values indicating more consistent performance.

**Table 13:** Most significant domain differences by model.

Model	Most Significant Domain Comparison	Difference (pp)	*p*-Value
ChatGPT-4o	No statistically significant differences	-	-
ChatGPT-o1	Diagnosis vs. Clinical examination	13.5	0.013
DeepSeek-R1	Diagnosis vs. Clinical examination	32.7	<0.001
Gemini-1.5-Pro	Diagnosis vs. Clinical examination	28.8	<0.001
Llama-3.1-405B	Diagnosis vs. Clinical examination	43.5	<0.001

Note: This table shows the most statistically significant performance difference between domains for each model.

**Table 14:** Accuracy by topic (percentage).

Topic (n, %)	ChatGPT-o1	ChatGPT-4o	DeepSeek-R1	Llama-3.1-405B	Gemini-1.5-Pro	Average
Small intestine disorders (19, 9.4%)	89.5	100.0	87.4	89.5	68.4	86.9
Biliary tract disorders (15, 7.4%)	93.3	90.7	86.7	58.7	60.0	77.9
Liver diseases (62, 30.5%)	86.8	85.5	77.1	84.8	76.8	82.2
Inflammatory bowel disease (28, 13.8%)	85.7	75.0	85.7	75.7	60.7	76.6
Oro-esophageal disorders (29, 14.3%)	82.1	**82.8**	82.8	79.3	65.5	78.5
Disorders of gut-brain interactions (6, 3.0%)	83.3	83.3	83.3	66.7	66.7	76.7
Stomach and duodenum conditions (15, 7.4%)	86.7	66.7	73.3	73.3	68.0	73.6
Large intestine pathologies (12, 5.9%)	66.7	66.7	78.3	68.3	83.3	72.7
Pancreatic disorders (12, 5.9%)	66.7	76.7	63.3	66.7	48.3	64.3
Bariatric conditions (5, 2.5%)	80.0	60.0	40.0	60.0	68.0	61.6
Overall (203, 100%)	84.0	81.7	79.0	77.2	68.5	78.1

Note: Bold values indicate the highest values for each topic.

**Table 15:** Best and worst topics by model.

Model	Best Topic (Accuracy %)	Worst Topic (Accuracy %)	Difference (% Points)	*p*-Value
ChatGPT-o1	Biliary tract disorders (93.3%)	Large intestine pathologies (66.7%)	26.7	<0.001
ChatGPT-4o	Small intestine disorders (100.0%)	Bariatric conditions (60.0%)	40.0	<0.001
DeepSeek-R1	Small intestine disorders (87.4%)	Bariatric conditions (40.0%)	47.4	<0.001
Llama-3.1-405B	Small intestine disorders (89.5%)	Biliary tract disorders (58.7%)	30.8	<0.001
Gemini-1.5-Pro	Large intestine pathologies (83.3%)	Pancreatic disorders (48.3%)	35.0	<0.001

## Data Availability

The questions used in this study were sourced from two commercial ESEGH preparation banks that are not publicly available. The model responses and analysis code are available from the corresponding author upon reasonable request.
